# Reduced efficacy of single-dose albendazole against *Ascaris lumbricoides*, and *Trichuris trichiura*, and high reinfection rate after cure among school children in southern Ethiopia: a prospective cohort study

**DOI:** 10.1186/s40249-024-01176-6

**Published:** 2024-01-22

**Authors:** Tigist Dires Gebreyesus, Eyasu Makonnen, Tafesse Tadele, Kalkidan Mekete, Habtamu Gashaw, Heran Gerba, Eleni Aklillu

**Affiliations:** 1 Department of Global Public Health, Karolinska Institutet, Karolinska University Hospital, Stockholm, Sweden; 2 Ethiopian Food and Drug Authority, Addis Ababa, Ethiopia; 3 Center for Innovative Drug Development and Therapeutic Trials for Africa, College of Health Sciences, Addis Ababa University, Addis Ababa, Ethiopia; 4 Departments of Pharmacology and Clinical Pharmacy, College of Health Sciences, Addis Ababa University, Addis Ababa, Ethiopia; 5 College of Medicine and Health Sciences, Hawassa University, Hawassa, Ethiopia; 6 Ethiopian Public Health Institute, Addis Ababa, Ethiopia

**Keywords:** Soil transmitted helminths, Neglected tropical diseases, Preventive chemotherapy, Albendazole, School age children, Ethiopia

## Abstract

**Background:**

Mass drug administration (MDA) program of albendazole to at-risk populations as preventive chemotherapy is the core public health intervention to control soil-transmitted helminths (STHs). Achieving this goal relies on drug effectiveness in reducing the parasite reservoirs in the community and preventing reinfection. We assessed the efficacy of albendazole against STH parasite infection and reinfection status after cure.

**Methods:**

A total of 984 schoolchildren infected with at least one type of STH parasite (hookworm*, Ascaris lumbricoides, Trichuris trichiura*) in southern Ethiopia were enrolled and received albendazole and praziquantel in MDA campaign conducted from January to March 2019. Stool exams at week-4 and at week-8 of post-MDA were done using Kato Katz technique. The primary outcome was efficacy assessed by cure rate (CR) and fecal egg reduction rates (ERRs) at four weeks of post-MDA. The secondary outcome was reinfection status defined as parasite egg positivity at eight weeks among those who were cured at 4 weeks of post-MDA. Group comparisons in CR and related factors were assessed with chi-square or Fisher’s exact tests. Predictors of CR were examined through univariate and multivariate regression analyses.

**Results:**

The overall CR and ERR for hookworm infection were 97.2% (95% *CI *94.6–99.4) and 97.02%, respectively. The overall CR and ERR for *A. lumbricoides* were 71.5% (95% *CI* 68.3–74.6) and 84.5% respectively. The overall CR and ERR and for *T. trichiura* were 49.5% (95% *CI *44.8–54.2) and 68.3%, respectively. The CR among moderate *T. trichiura* infection intensity was 28.6%. Among children cured of hookworm, *A. lumbricoides* and *T. trichiura* at week 4 post-MDA, 4.6%, 18.3% and 52.4% became reinfected at week*-*8 post-MDA, respectively. Significantly lower CR (36.6%) and higher reinfection after cure (60.6%) among *A. lumbricoides* and *T. trichiura* coinfected children than *A. lumbricoides* only (CR = 69.6%, reinfection rate = 15.1%) or *T. trichiura* only infected children (CR = 55.6%, reinfection rate = 47.1%) was observed. Pre-treatment coinfection with ≥ two types of STH parasites was significantly associated with re-infection after cure.

**Conclusion:**

Albendazole MDA is efficacious against hookworm but has reduced efficacy against *A. lumbricoides* and is not effective against *T. trichiura*. The low drug efficacy and high reinfection rate after cure underscore the need for alternative treatment and integration of other preventive measures to achieve the target of eliminating STHs as a public health problem by 2030.

**Graphical Abstract:**

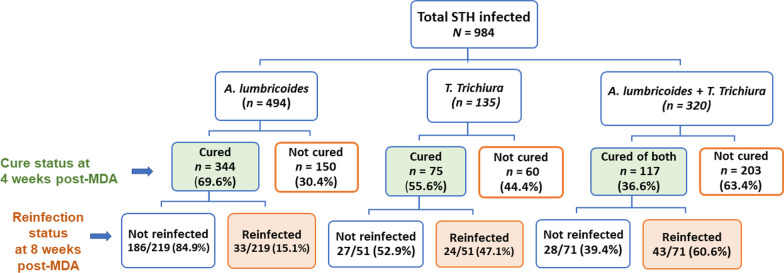

## Background

Soil-transmitted helminths (STHs) infection are one of the most prevalent neglected tropical diseases (NTDs), with an estimated 1.5 billion people infected globally, primarily in Africa, America, and Asia [1]. STH parasites mainly affect marginalized and deprived communities in low- and middle-income countries with poor access to clean water, sanitation, and hygiene. The three most common parasites infecting humans are *A. lumbricoides*, *T. trichiura* and hookworm [1]. Currently, nearly a quarter (24%) of the world’s population living in 101 countries is estimated to be infected with these parasites [1, 2]. Sub-Saharan Africa (SSA) harbors the highest burden of STH infection globally. In Ethiopia, approximately 88.1 million people live in STH endemic areas [3]. Among populations at risk, school-aged children represent a significant proportion, accounting for nearly one-third (27.9 million). A recent systematic review reported that more than one-third of Ethiopian school-age children are infected with STH [4]. Children are at increased risk of repeated infections by STHs due to their frequent exposure to contaminated soil and poor hygiene and sanitary practices. Chronic and repeated infections by STHs among children result in serious health impacts causing malnutrition, impaired physical growth, and retarded intellectual development [1].

The WHO endorsed a resolution on the STH control strategy in 2001 through the delegates of the World Health Assembly, mainly targeting periodic treatment of the population at risk living in endemic areas aiming at reducing worm burden and hence reducing morbidity [5]. The WHO targeted treating at least 75% of children living in endemic countries. The current WHO guidelines recommending large-scale anthelminthic drugs for preventive chemotherapy against STHs are albendazole or mebendazole once (where prevalence is > 20%) and twice a year (where prevalence is > 50%) based on the prevalence of STHs [6]. In addition to MDA, improving access to clean water, hygiene, and sanitation (WASH) is recommended for reducing transmission and reinfection.

Following the WHO strategic plan for the control and elimination of STHs, endemic countries have been implementing periodic mass drug administration (MDA) in the form of preventive chemotherapy. The increased donor funding and drug donations allowed addressing a large amount of the population at risk through preventive chemotherapy. Initially, the WHO targeted eliminating STHs as a public health problem (defined as < 1% prevalence of moderate to heavy infection intensity among at-risk populations) by 2020 [7]. Despite the implementation of periodic large-scale PC for the last two decades with high coverage, many endemic countries, including Ethiopia, did not achieve the initial WHO target of eliminating STHs by 2020 [2, 8–10]. In 2021 alone, 511 million children received PC against STHs in endemic countries [7]. The revised WHO target for 2030 mainly focused on morbidity control, which aimed at reducing the prevalence of moderate to heavy infection intensity to < 2% among preschool and school-aged children [11].

Untreated infected individuals can serve as a parasite reservoir for continued transmission in the community and treating all populations at risk through MDA at regular intervals kills the parasite in infected individuals, leading to reduced transmission and prevention of disease. Repeated rounds of MDA have successfully reduced the disease burden over time, especially moderate and heavy infections. However, as parasite drug exposure increases, the risk of parasite tolerance and the emergence of drug resistance remains a concern. Indeed, recently, several studies reported decreased efficacy of albendazole against STHs, particularly in an area with high drug pressure [12–16]. Although the WHO recommends member states and NTD programs to monitor the efficacy of anthelminthic drugs used in MDA in cases of suspected treatment failure or when preventive chemotherapy is implemented for four or more years irrespective of drug failure [17], this recommendation is not implemented by NTD programs in resource-limited countries.

Several studies have assessed the efficacy of drugs used in MDA, but the results vary between the type of STH parasite, study population, history of preventive chemotherapy and geographic location. STH infections are distributed widely throughout Ethiopia. Decreased efficacy of mebendazole and albendazole in areas with repeated preventive chemotherapy in Northwest Ethiopia has been reported [18, 19]. In particular, reduced efficacy of albendazole against *Trichuris trichiura* was reported in previous studies, including systematic reviews and meta-analyses [12, 15, 20–24]. Currently, the control and elimination of STHs mainly relies on benzimidazoles only, and preserving their effectiveness is crucial while investing in new treatment options. Therefore, integrating efficacy surveillance in the national MDA program is necessary. The national NTD program of Ethiopia emphasized the need for post-MDA surveys and impact assessments; however, the efficacy of albendazole has not been monitored despite several rounds of school-based MDA implemented since 2007 and data on reinfection status after cure is lacking [25]. Therefore, after a decade-long annual MDA program implementation, we conducted large-scale efficacy surveillance of single-dose albendazole among STH-infected school-aged children living in two rural districts in southern Ethiopia and re-infection status after cure.

## Methods

### Study design, settings, and population

This prospective, observational efficacy surveillance of single dose albendazole given through MDA campaign was conducted from January to March 2019. The study populations were MDA eligible and STH-infected school children aged 5–15 years attending four primary schools in two rural districts (Hawella Tula and Wondo Gennet) in southern Ethiopia. According to the Ethiopian national NTD mapping conducted between 2013 and 2014, the two study districts are schistosomiasis and STHs co-endemic and are classified as high-prevalence communities with > 70% prevalence of STHs [26]. The targeted population group for preventive chemotherapy are school-aged children and biannual preventive chemotherapy has been implemented since then as per the WHO recommendation [27], and the last preventive chemotherapy was in May 2018.

Two weeks prior to the planned school-based MDA, eligible children attending the four study schools were screened for STH infection [10]. Stool samples were collected for microscopic examinations to screen and diagnose STHs. The eligibility criteria for enrollment were STH-infected children attending the study schools and whose parents or primary caregivers gave written consent. Nine hundred eighty-four children infected with at least one type of STH parasite (hookworms, *A. lumbricoides*, *T. trichiura*) were enrolled in the current albendazole efficacy study.

### Preventive chemotherapy and follow-up

All children attending the four primary schools received single dose albendazole 400 mg and praziquantel based on the height of the children (≥ 94 cm dose pole, designed to deliver a dose of at least 40 mg/kg) through the MDA program under direct observation following the WHO guidelines [27, 28]. The school-based preventive chemotherapy in the study districts was implemented by the Ethiopian National NTD program. The study team had no role in the planning or implementation of the MDA. A follow-up stool sample was collected at week 4 of post-MDA from STH-infected study participants to assess albendazole efficacy following the WHO guidelines [17]. STH-infected children were followed-up to assess anthelmintic drug efficacy at week-4 of post MDA. An additional follow up sample was collected at week 8 of post-MDA from study participants who were cured at week 4 of post-MDA to assess reinfection status.

### Screening for STH parasite species

Stool samples collected at baseline for diagnosis, at week 4 for assessment of drug efficacy and at week 8 for assessment of reinfection status were processed and analyzed using the Kato-Katz technique. Two thick smear slides were prepared from each stool sample using 41.7 mg template, and the slides were read immediately for hookworm microscopic examination [29]. For the rest of the parasites, the slides were kept for an hour to ensure clear visibility. A quality assurance test was performed by senior laboratory professionals by re-examining 10% of the samples. For each participant, the average mean egg count of the two slides was multiplied by a factor of 24 to calculate the egg per gram of stool (epg) [29]. The pre-treatment infection intensity was classified as light, moderate and heavy for each parasite following the WHO reference [30]; *T. trichiura* [Light (1–999 epg), moderate (1000–9999 epg), heavy (≥ 10,000 epg)]; *A. lumbricoides* [Light (1–4999 epg), Moderate (5000–49,999 epg), Heavy (≥ 50,000 epg)]; Hookworm [Light (1–1999 epg), Moderate (2000–3999 epg), Heavy (≥ 4000 epg)];

### Study outcomes

The primary study outcome was efficacy based on the thick smear Kato‐Katz method at 4 weeks post‐treatment assessed by parasitological cure rate (CR), defined as the proportion of children who were egg positive at baseline and became egg negative, and the egg reduction rate (ERR) calculated as follows: 100 times [1 − (Arithmetic mean of epg after treatment/Arithmetic mean of epg before treatment)], as recommended by the WHO [17]. The secondary outcome was reinfection (egg positivity) status, defined as the proportion of children who were egg negative at week 4 but became egg positive at 8 weeks post-MDA for each STH parasite.

### Statistical analysis

Sociodemographic, clinical, and medical histories, any comorbidities, and nutritional status (assessed using anthropometric data), were collected using a case record format, entered, cleaned in an Excel spreadsheet. Data were analyzed using IBM SPSS Statistics for Windows software, Version 24.0 (IBM Corp, Armonk, NY, USA). The test differences between groups were compared using the chi-square test. Associations between cure rates and independent categorical variables were analyzed using the chi-square test or Fisher’s exact test. Predictors of cure rate were analyzed using univariate followed by multivariate regression analysis. Predictor variables with *P* ≤ 0.2 in the univariate analyses were entered into the multivariate analysis. *P* values < 0.05 were considered statistically significant.

### Ethical consideration

This study was conducted as per the Declaration of Helsinki and obtained ethical approval from regional (Ref No: 902-6-19/14666) and national (Ref No: MoSHE 11RD/141/9848/20) ethics review committees. In addition, permission was granted from relevant regional, zonal, and woreda health and education offices. Prior to enrollment, study participants and their guardians/parents were informed about the study objective, significance, and data collection procedures. School children whose parents/guardians gave written informed consent were enrolled in the study. For children aged > 12 years, both written informed consent from the parent or guardian and assent from the study participant was obtained.

## Results

### Sociodemographic and baseline characteristics

A total of 984 schoolchildren aged 5–15 years who were infected by at least one type of STH parasite were enrolled in this study. Approximately half (49.8%) of the participants were male, and 74.0% were above the age of nine. Approximately one-third (30.4%) of the participants were coinfected with *Schistosoma mansoni*. Most of the participants were infected with *A. lumbricoides* (82.7%), followed by *T. trichiura* (46.2%) and hookworm (11.1%). *A. lumbricoides* infected children with light infection intensity were 67.7%. There was no heavily infected child with *Trichuris trichiura*, and the majority (96.9%) of infected children had light infection intensity. All participants infected with hookworms had light infection intensity (Table 1). Among the *A. lumbricoides*-infected school children, 39.3% were coinfected with *Trichuris trichiura*.

**Table 1:** Sociodemographic and baseline characteristics of the study participants

Variables	Category		Number	Percentage
Sex	Male		490	49.8
	Female		494	50.2
Age category	≤ 9 years		256	26
	≥ 10 years		728	74
District	Hawela Tula		436	44.3
	Wondo Gennet		548	55.7
Study schools	Bushulo primary school		289	29.4
	Kidus pawulos primary school		91	9.2
	Finchawa primary school		56	5.7
	Wosha primary school		548	55.7
Coinfection with Schistosoma mansoni	No		685	69.6
	Yes		299	30.4
Type of STH parasite coinfections	Singel type parasite infection		614	62.4
	Dual parasite infection		340	34.6
	Triple parasite infection		30	3
*Hookworm * (n = 109)	Infection intensity	Light intensity	109	100
		Moderate intensity	0	0
		Heavy intensity	0	0
	Coinfection with other type of STH parasites	No	23	21.1
		Yes	86	78.9
*Ascaris lumbricoides * (n = 814)	Infection intensity	Light intensity	550	67.6
		Moderate intensity	240	29.5
		Heavy intensity	24	2.9
	Coinfection with other type of STH parasites	No	347	42.6
		Yes	467	57.4
*Trichuris trichiura* (n = 455)	Infection intensity	Light intensity	441	96.9
		Moderate intensity	14	3.1
		Heavy intensity	0	0
	Coinfection with other type of STH parasites	No	80	17.6
		Yes	375	82.4

*STH* Soil-transmitted helminth

### Parasitological cure rate and associated factors

**Fig. 1:**
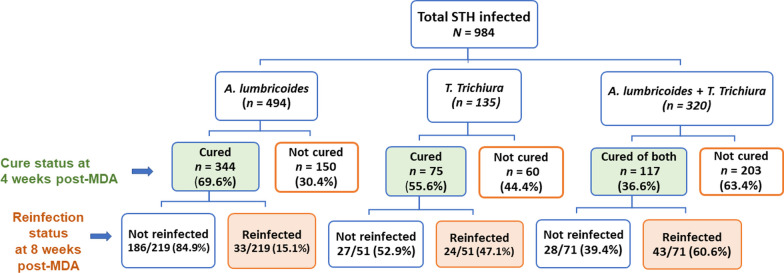
Study flowchart: cure rates for *Ascaris lumbricoides*, *Trichuris trichiura*, and their confections at 4 weeks of post albendazole mass drug administration (MDA) and reinfection status at eight weeks of post MDA. *STHs* soil-transmitted helminths

Overall, 46% of the study participants were cured of any type of STH infection at week 4 of post MDA. The observed cure rate in comparison with the WHO efficacy threshold for each parasite at week 4 follow-up is presented in Table 2. The majority (97.2%) of hookworm-infected children were cured at week-4 of post-MDA. The cure rate for *A. lumbricoides* was 71.5% at week 4 post-MDA follow-up, which is below the WHO threshold (≥ 95%) for efficacy. The cure rate for *T. trichiura* was 49.5% at the week 4 follow-up. Like *A. lumbricoides,* the cure rates for *T. trichiura* were lower than the WHO threshold (≥ 50%) for the efficacy of albendazole in the treatment of *Trichuris trichiura* infection. The cure rate was very low among the coinfected children, and only 36.6% of coinfected were cured for both *A. lumbricoides* and* T. trichiura* (Fig. 1).

**Table 2:** Cure rates at week 4 of albendazole mass drug administration

Types of STH infection			Week 4 post MDA			WHO reference threshold (%)
			Total *n*	Number cured	*CR* (95%* CI*), %	
Hookworm	All (light infections)		109	106	97.2 (92.2–99.4)	≥ 95
*Ascaris lumbricoides*	Overall		814	582	71.5 (68.3–74.6)	≥ 95
	Infection intensity	Light	550	404	73.5 (69.6–77.1)	
		Moderate	240	161	67.1 (60.8–73.0)	
		Heavy	24	17	70.8 (48.9–87.4)	
*Trichuris trichiura*	Overall		455	225	49.5 (44.8–54.2)	≥ 50
	Infection intensity	Light	441	221	50.1 (45.4–54.9)	
		Moderate	14	4	28.6 (8.4–58.1)	

*CR* Cure rate

The factors associated with the cure rate and their predictors for the three parasite infections at week-4 are presented in Tables 3 and 4. The cure rate for hookworm infection was not significantly associated (*P* > 0.05) with the baseline sociodemographic characteristics of the study participants. The cure rate for *A. lumbricoides* was significantly associated with the age group of study participants, in which the lower age groups (≤ 9 years) had a lower cure rate than the higher age groups (*P* = 0.006). Although not statistically significant (*P* = 0.11), the cure rate for *T. trichiura* infection was lower among moderately infected than lightly infected school children (28.6% versus 50.1%).

**Table 3:** Factors associated with cure rates for each STH infections at week 4 post single dose albendazole mass drug administration

Variables	Hookworm			*Ascaris lumbricoides*			*Trichuris trichiura*		
	Total *n*	Cure rate (%)	*P*	Total *n*	Cure rate (%)	*P*	Total *n*	Cure rate (%)	*P*
Age group									
≤ 9 years	33	100	0.25	225	64.4	0.006	107	46.7	0.52
≥ 10 years	76	96.1		589	74.2		348	50.3	
Sex									
Male	63	96.8	0.75	403	71.2	0.86	219	47.5	0.42
Female	46	97.8		411	71.8		236	51.3	
Coinfection with *Schistosoma mansoni*									
No coinfection	70	95.7	0.19	576	70.8	0.51	288	49.3	0.94
With coinfection	39	100		238	73.1		167	49.7	
Number of STH infections at baseline									
Single infection	34	94.1	0.28	456	69.5	0.29	127	54.3	0.15
Double infection	45	100		329	73.6		299	48.8	
Triple infection	30	96.7		29	79.3		29	34.5	
Coinfection with other STH parasites									
Yes	86	98.8	0.05	467	73.7	0.11	375	49.6	0.89
No	23	91.3		347	68.6		80	48.8	
Pretreatment infection intensity									
Light	109	97.2		550	73.5	0.19	441	50.1	0.11
Moderate				240	67.1		14	28.6	
Heavy				24	70.8				

*STH* soil-transmitted helminth

**Table 4:** Predictors of cure rate at week 4 of single dose albendazole mass drug administration

Variables	Hookworm			*Ascaris lumbricoides*					*Trichuris trichiura*				
	Cured *n* (%)	c*OR* (95% *CI*)	*P*	Cured *n* (%)	c*OR* (95% *CI*)	*P*	a*OR* (95% *CI*)	*P*	Cured *n* (%)	c*OR* (95% *CI*)	*P*	a*OR* (95% *CI*)	*P*
Age group													
≤ 9 years	33 (100)	Omitted		145 (64.4)	1	0.006	1	0.006	50 (46.7)	1	0.52		
≥ 10 years	73 (96.1)			437 (74.2)	1.59 (1.14–2.21)		1.6 (1.14–2.22)		175 (50.3)	1.15 (0.75–1.78)			
Sex													
Male	61 (96.8)	1.5(0.13–16.78)	0.75	287 (71.2)	1	0.86			104 (47.5)	1	0.42		
Female	45 (97.8)	1		295 (71.8)	1.03 (0.76–1.39)				121 (51.3)	1.16 (0.81–1.68)			
Co-infection with *Schistosoma mansoni*													
No co-infection	67 (95.7)	Omitted		408 (70.8)	1	0.51			142 (49.3)	1	0.94		
With co-infection	38 (100)			174 (73.1)	1.12 (0.8–1.57)				83 (49.7)	1.02 (0.7–1.5)			
Number of STH infections at baseline													
Single infection	32 (94.1)	Omitted		317 (69.5)	0.6 (0.24–1.5)	0.27			69 (54.3)	2.3 (0.97–5.24)	0.06	2.3 (0.98–5.31)	0.06
Double infection	45 (100)			242 (73.6)	0.73 (0.3–1.84)	0.50			146 (48.8)	1.81 (0.82–4.03)	0.14	1.81(0.81–4.02)	0.15
Triple infection	29 (96.7)			23 (79.3)	1				10 (34.5)	1			
Co-infection with other STH parasites													
Yes	85 (98.8)	0.12(0.01–1.42)	0.09	344 (73.7)	1	0.11	1	0.10	186 (49.6)	1	0.89		
No	21 (91.3)	1		238 (68.6)	0.78 (0.58–1.06)		0.77(0.57–1.05)		39 (48.8)	0.97 (0.6–1.6)			
Pre-treatment infection intensity													
Light	106 (97.2)			404 (73.5)	1.14 (0.46–2.80)	0.78			221 (50.1)	2.5 (0.78–8.13)	0.12	2.27 (0.8–8.4)	0.12
Moderate				161 (67.1)	0.84 (0.33–2.11)	0.71			4 (28.6)	1		1	
Heavy				17 (70.8)	1								

*n*: Total number of participants within each category; 1: reference category; a*OR*: Adjusted odds ratio; c*OR*: Crude odds ratio, *CI *Confidence interval STHs: Soil transmitted helminths. Predictor variables with P ≤ 0.2 in the univariate analyses were entered into the multivariate analysis

### Egg reduction rates

The egg reduction rates for the three parasites were assessed at the week 4 follow-up in comparison with the WHO threshold for efficacy of albendazole (Table 5). The observed egg reduction rate for hookworm infection of 98.8% was above the WHO threshold (≥ 90%). On the other hand, the egg reduction rate for *A. lumbricoides* at the week 4 follow-up of 84.5% was below the WHO threshold (≥ 95%) for the efficacy of albendazole. However, the egg reduction rate for *A. lumbricoides* in heavily infected children (98.9%) was above the WHO threshold. Unlike the cure rate, the egg reduction rate for *T. trichiura* at week 4 follow-up was 68.3%, which is above the WHO threshold (≥ 50%).

**Table 5:** Egg reduction rates for Hookworm, *Ascaris lumbricoides* and *Trichuris trichiura* infection with WHO thresholds

Infection intensity	Hookworm			*A. lumbricoides*			*T. trichiura*		
	Total *n*	ERR (%)	WHO reference threshold	Total *n*	ERR (%)	WHO reference threshold	Total *n*	ERR (%)	WHO reference threshold
Light	109	98.8	≥ 90%	550	40	≥ 95%	441	72.1	≥ 50%
Moderate	0	0		240	87.3		14	61.2	
Heavy	0	0		24	98.9		0	0	
Overall	109	98.8		814	84.5		455	68.3	

*ERR,* egg reduction rate

### Reinfection status after being cured

At the week 8 follow-up, we assessed reinfection status of the three parasites after being cured at 4 weeks of post MDA (Table 6); 65 hookworm-, 367 *A. lumbricoides* and 143 T*. trichiura*-infected children who were cured at week-4 of post MDA were present for the 8 weeks follow-up. Among the 65 hookworm-infected children who were cured at 4-weeks of treatment, 62 (94.4%) remained egg free at the week 8 follow-up. Three participants who were cured at week 4 became egg positive (reinfected) at the week 8 follow-up, showing 4.6% reinfection after cure. Among the 367 *A. lumbricoides* infected children who were cured at 4-weeks of treatment, only 300 (81.7%) remained egg free at the week 8 follow-up; and 67 (18.3%) of the participants became reinfected at week 8 post-MDA. Among the 143 T*. trichiura* infected children who were cured at 4-weeks of treatment, only 68 (47.6%) of the participants remained egg negative at week 8 follow-up, and 75 (52.4%) of participants became reinfected at week 8 post MDA follow-up.

**Table 6:** Reinfections for Hookworm, *Ascaris lumbricoides* and *Trichuris trichiura* infection at week 8 of single dose albendazole mass drug administration

Category	Hookworm		*A. lumbricoides*		*T. trichiura*	
	Number	Percent	Number	Percent	Number	Percent
Cured at weeks 4 and remained egg free at week 8 of post MDA	62	95.4	300	81.7	68	47.6
Cured at week 4 but become egg positive at week 8 (reinfected)	3	4.6	67	18.3	75	52.4

For *A. lumbricoides-* and *T. trichiura*-reinfected participants, we also assessed the possible factors associated with reinfection (Table 7). For *A. lumbricoides*, schools and coinfection with other STHs were significantly associated with reinfection (*P* = 0.001 and 0.005, respectively). Although not statistically significant, participants coinfected with other STHs had higher reinfection for *T. trichiura* compared those who had only *T. trichiura* infection (55.2% versus 40.7%).

**Table 7:** Factors associated with reinfection for *Ascaris lumbricoides* and *Trichuris trichiura* at week 8 of single dose albendazole mass drug administration

Factors	Category	*A. lumbricoides*			*T. trichiura*		
		Total *n*	Reinfected (%)	*P* value	Total *n*	Reinfected (%)	*P* value
Age group	≤ 9 years	89	16.9	0.76	24	62.5	0.37
	≥ 10 years	278	18.7		119	50.4	
Sex	Male	187	17.1	0.59	71	57.7	0.24
	Female	180	19.4		72	47.2	
School	Bushulo	128	9.4	0.001	45	42.2	0.23
	Finchawa	11	9.1		2	100	
	K. Pawulos	50	14.0		15	53.3	
	Wosha	178	26.4		81	56.8	
Pretreatment infection intensity	Light	255	18.0	0.54	140	51.4	0.25
	Moderate	99	20.2		3	100	
	Heavy	13	7.7		0	0	
Coinfection with other STH parasites	Yes	223	22.9	0.005	116	55.2	0.20
	No	144	11.1		27	40.7	

## Discussion

The WHO recommends assessing the efficacy of anthelminthic drugs used for preventive chemotherapy when reduced efficacy is observed or when the PC program is implemented for more than four consecutive years [17]. Recently we reported that 54.7% of school children in the same study school had at least one type of STH parasite infection in the study districts despite multiple rounds of preventive chemotherapy implemented by the national NTD programs [10]. Several factors can contribute to the persistently high prevalence of STH infection despite several rounds of MDA with high coverage. As the drug pressure increases, parasite tolerance or resistance becomes a concern. We conducted this follow-up study to assess the efficacy of albendazole MDA among infected children as well as the status of reinfection after cure in the same study schools.

Our findings revealed that albendazole is effective against hookworms, with a high cure and egg reduction rates. The observed cure rate (97.2%) was above the WHO-recommended threshold for efficacy. Similarly, the ERR 98.8% observed was also above the WHO threshold. The high cure rate and ERR observed for hookworms indicate that albendazole can significantly reduce disease burden by decreasing infection intensities.

In contrast to the efficacy observed for hookworms, we found a reduced efficacy against *A lumbricoides* with a low egg reduction rate. Similarly, the cure rate observed for *A. lumbricoides* was below the WHO threshold recommended for efficacy. A similar decreased efficacy of albendazole against *A. lumbricoides* was also reported in previous studies [16, 31]. The cure rate of albendazole against *A. lumbricoides* was significantly associated with the age group of participants, where lower age groups had a low cure rate. However, albendazole treatment resulted in significant egg reduction among heavily infected children, indicating that the drug could result in a significant reduction in the infection intensity among heavily infected children, although it failed to clear the infection, especially in lightly infected children. These uncured children might become a reservoir of infection and continue to transmit the disease after treatment. Recently our study group also reported a reduced efficacy of albendazole given through MDA for the treatment of *A. lumbricoides* infected children in Rwanda [12]. In our week 8 follow-up post-MDA, we observed a significant number of reinfections (18.3%) of *A. lumbricoides*. Therefore, to prevent reinfection among treated and cured children and achieve the global target of eliminating STHs as a public health problem by 2030, the implementation of other strategies, such as clean water supply, sanitation, and hygiene (WASH), to avoid contamination of the environment is very important in addition to preventive chemotherapy.

Our results showed that albendazole has low cure and egg reduction rates for the treatment of *T. trichiura*. In addition, more than half of the cured children (52.4%) became reinfected within a month after being cured. The progressive significant decrease in efficacy of against *T. trichiura* over the past two decades is well recognized. Previous similar studies, including a systematic review and meta-analysis, also reported that albendazole is not efficacious against *T. trichiura* infection in different settings [12, 15, 20–24]. The wider use of albendazole in MDA as preventive chemotherapy to control STH and lymphatic filariasis may cause high drug pressure on parasites and may trigger drug resistance. This finding calls for a concerted effort to search for a better alternative treatment against *T trichiura* infection. Few clinical trials that explored alternative combination treatments regimens for *T. trichiura* infection reported conflicting results with three studies, including systematic reviews and meta-analyses, reported a promising outcome [32–34], and one recent study reported no significant improvement with drug combinations [35]. Our study finding is in line with previous reports including a systematic review and meta-analysis that reported a significant rapid reinfection after treatment for* A. lumbricoides* and *T. trichiura,* which clearly indicates the importance of integrating preventive chemotherapy with other STH prevention strategies for preventing reinfection [36, 37].

The observed reduced efficacy of mass albendazole chemotherapy against *T. trichiura* and *A. lumbricoides* in Ethiopian school children noted in this study and elsewhere in Africa raises concerns [16]. If anthelminthic treatment with broad coverage demonstrates a low impact on the target parasite, suspicion of drug resistance should arise [17]. Indeed, recent studies reported benzimidazole resistance-associated with single nucleotide polymorphism in the beta-tubulin gene in *A. lumbricoides*, and some of the mutations detected at high frequencies in Haiti, Kenya, and Panama where benzimidazoles is extensively used [38, 39]. Therefore, molecular studies are imperative to evaluate potential drug resistance and further epidemiological studies to evaluate how widespread it is in endemic areas where the drug is used widely. As STH control initiatives depend on a limited range of benzimidazole anthelmintics, early identification of potential parasite resistance and follow up mitigation strategies, including the exploration of combination therapy to extend the effectiveness of current anthelmintic drugs, should be considered [23]. In addition, efforts to intensify the search for new anthelmintic drugs or intervention measures are imperative.

This study has some limitations. In this study, we used the Kato-Katz technique, which is a WHO-recommended method for assessing the efficacy of anthelminthic drugs. However, the Kato-Katz is less sensitive in low infection intensities [40], which may overestimate the efficacy result, especially for hookworm and *T. trichiura,* which had a high number of participants with low infection intensities at baseline assessment [10]; hence, we consider this as a limitation to our study.

## Conclusions

Single dose albendazole given through school-based MDA campaign as preventive chemotherapy is effective against hookworm, has reduced efficacy against *A. lumbricoides* and is not effective against *T. trichiura* infections. The reduced efficacy observed in our study, especially against *A. lumbricoides* and *T. trichiura* along with persistently high prevalence of STH in our study area might be an indication of emerging resistance after multiple rounds of preventive chemotherapy. Furthermore, the observed high reinfection after cure underscores the need to integrate other preventive measures such as WASH in addition to efforts to develop new effective anthelmintic drugs to control *A. lumbricoides* and *T. trichiura* infections. Ethiopia has targeted to eliminate the transmission of STHs by 2030 [41]. However, with the current albendazole based MDA intervention strategy, the global target and milestone set to control and eliminate STHs as a public health problem by 2030 may not be achievable without availing alternative treatment options and combining them with other control and elimination strategies.

## Data Availability

All data generated or analyzed during this study are included in the manuscript.

## Abbreviations

MDA: Mass Drug Administration
NTD: Neglected tropical diseases
PC: Preventive chemotherapy
SSA: Sub-Saharan Africa
STH: Soil-transmitted helminths
WHO: World Health Organization
a*OR*: Adjusted odds ratio
c*OR*: Crude odds ratio
*CI *: Confidence interval
CR: Cure rate
ERR: Egg reduction rate
WASH: Water, sanitation and hygiene
